# Substrate Mediated Enzyme Prodrug Therapy

**DOI:** 10.1371/journal.pone.0049619

**Published:** 2012-11-13

**Authors:** Betina Fejerskov, Alexander N. Zelikin

**Affiliations:** 1 Department of Chemistry, Aarhus University, Aarhus, Denmark; 2 iNano Interdisciplinary Nanoscience Centre, Aarhus University, Aarhus, Denmark; RMIT University, Australia

## Abstract

In this report, we detail Substrate Mediated Enzyme Prodrug Therapy (SMEPT) as a novel approach in drug delivery which relies on enzyme-functionalized cell culture substrates to achieve a localized conversion of benign prodrug(s) into active therapeutics with subsequent delivery to adhering cells or adjacent tissues. For proof-of-concept SMEPT, we use surface adhered micro-structured physical hydrogels based on poly(vinyl alcohol), β-glucuronidase enzyme and glucuronide prodrugs. We demonstrate enzymatic activity mediated by the assembled hydrogel samples and illustrate arms of control over rate of release of model fluorescent cargo. SMEPT was not impaired by adhering cells and afforded facile time - and dose – dependent uptake of the in situ generated fluorescent cargo by hepatic cells, HepG2. With the use of a glucuronide derivative of an anticancer drug, SN-38, SMEPT afforded a decrease in cell viability to a level similar to that achieved using parent drug. Finally, dose response was achieved using SMEPT and administration of judiciously chosen concentration of SN-38 glucuronide prodrug thus revealing external control over drug delivery using drug eluting surface. We believe that this highly adaptable concept will find use in diverse biomedical applications, specifically surface mediated drug delivery and tissue engineering.

## Introduction

Hydrogel biomaterials find extensive use in biotechnology and biomedicine as matrices for controlled drug release, cell culture and tissue engineering. [1]–[8] With appropriate modification, virtually any natural or synthetic water-soluble polymer can be used as a gel-forming material, an aspect which spells a unique diversity of properties available through the choice of constituting polymers. [2] A further unique opportunity in the design of biomaterials using hydrogels is that through judicious choice of crosslinking density it becomes possible to vary the characteristics of the final hydrogel, specifically water content, diffusivity of substrates through the matrix, [3] Young’s modulus of the material etc., [9] and mimic the properties of soft human tissues. [10] However, a persistent limitation of hydrogels relates to opportunities in engineering retention and controlled release of therapeutic cargo, specifically low molecular weight solutes. [7] In contrast to e.g. solid matrices comprised of slowly degrading organic polymers, [11] hydrogels are highly hydrated and present a weak barrier for diffusion of cargo from the hydrogel to the external environment. Further, these materials are typically characterized by a significant “burst release”, i.e. un-controllable loss of cargo upon hydration and swelling of the matrix. As a partial solution to these shortcomings, cargo can be associated with the hydrogel through ionic interaction, [7] specifically for hydrogels comprised of ionic polymers and counter-charged cargo, yet this strategy suffers from non-specific loss of payload in physiological milieu with its associated high ionic strength. Affinity – based immobilization is a nature inspired approach [12] yet it is largely inapplicable to small solutes. Drug molecules can be covalently linked to the matrix; [3] however, this strategy is limited by synthetic opportunities associated with each polymer and drug candidate. Arguably the most advanced design is that of “composite hydrogels”, [7], [13] i.e. hydrogel matrices with embedded nanoparticles, liposomes or another type of drug reservoir, in which case the latter provides for a controlled drug release performance of the biomaterial. This strategy was successfully implemented in diverse hydrogel systems for a range of biomedical applications [7] such as drug releasing contact lenses. [14] Nevertheless, despite significant level of development, hydrogels still largely fail to answer a continuous call for advanced opportunities in control drug release engineered into matrices for soft tissue engineering. [15], [16].

**Figure 1:**
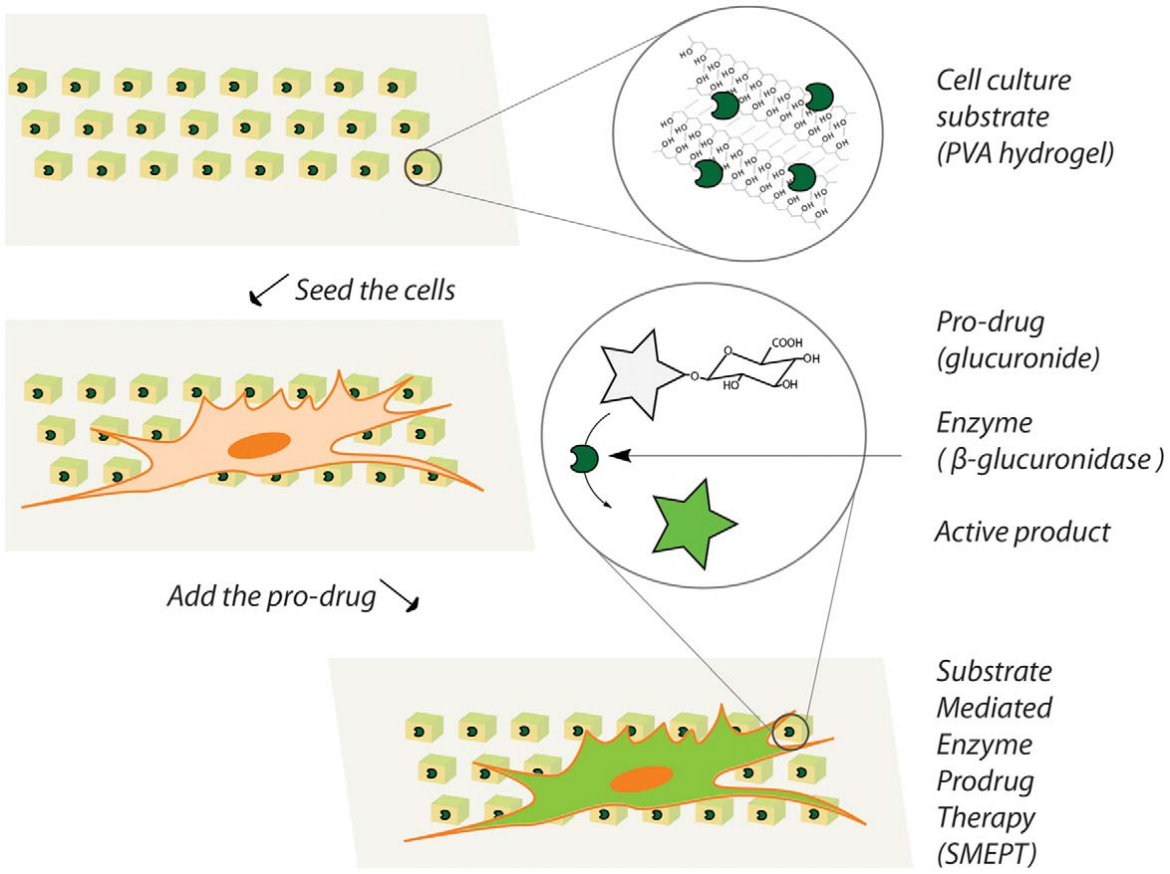
Schematic illustration of the concept of Substrate Mediated Enzyme Prodrug Therapy (SMEPT). A cell culture substrate contains an enzyme for conversion of an inactive prodrug into an active product which is then internalized by the adhering cells or adjacent tissues. SMEPT affords both, an amplification of the deliverable payload and an interactive adjustment of the dose and the rate of drug release.

In an effort to address this issue, we develop a novel method in drug delivery mediated by hydrogel matrices, namely Substrate Mediated Enzyme Prodrug Therapy (SMEPT). Specifically, we propose to use hydrogel matrices for immobilization of enzymes and accomplish localized conversion of externally added benign prodrugs into active therapeutics for presentation to adhering of adjacent cells and tissues, Figure 1. Enzyme immobilization into hydrogel matrices is well documented for e.g. biomass conversion [17], [18] and delivery of therapeutically active protein cargo, [19], [20]. From a different perspective, enzyme/prodrug immobilization into materials has also been achieved towards creation of mechanically activated production of cargo. [21], [22] However, to the best of our knowledge, there are no prior examples of in situ enzymatic generation of therapeutic cargo within a hydrogel phase as a platform for controlled drug delivery.

As matrices for SMEPT, we use micro-structured (µS), micrometer-thick surface adhered physical hydrogels[23]–[25] based on a polymer with decades of biomedical prominence, poly(vinyl alcohol), PVA. [26], [27] The choice of the substrate was driven by the following criteria: PVA hydrogels are well characterized materials in tissue engineering, [5], [28] yet suffer from poor control over drug immobilization and release [7], [26] and would therefore tremendously benefit from engineered opportunities in controlled drug delivery. [15] Further, µS and micro-patterned substrates are pivotal in (co)culture of mammalian cells towards 2D and 3D reconstruction of organs and tissues. [8], [29]–[31] Surface-adhered substrates have also recently gained recognition as powerful tools in surface mediated drug delivery for e.g. prevention of restenosis and facilitated acceptance of implants. [32] Finally, surface adhered nature allows using a host of techniques for visualization and characterization of the substrate. [24].

In our previous reports, [23], [24], [33] we characterized in detail assembly of µS PVA hydrogels via micro-transfer molding, i.e. topography replication using poly(dimethylsiloxane), PDMS, stamps. Solutions of PVA were used to fill the cavities in PDMS stamps and the latter were then clamped at finger tight pressure using glass cover slips. Initial hydrogelation of the polymer within the cavities and adhesion to the surface of the coverslip produced surface-adhered PVA materials. We developed non-cryogenic techniques to afford subsequent polymer hydrogelation, specifically using coagulating kosmotropic salt, sodium sulfate, aqueous isopropanol or oligomeric (liquid) polyethylene glycol. Resulting µS hydrogels were typically characterized with a height of ∼1 µm and lateral dimensions programmed via a design of PDMS stamp. We demonstrated flexibility of this platform as a tool for a rational design of intelligent biointerfaces with control over surface topography [24] and cell adhesive properties [23], [13] as well as mechanical properties of the matrix (Young’s modulus). [23], [24], [33].

With regard to the choice of an enzyme, design of SMEPT benefits from the knowledge on a well-characterized biomedical platform, namely “antibody directed enzyme prodrug therapy” (ADEPT). [34]–[36] In this drug delivery opportunity, enzyme is anchored within the human body at the target site (e.g. tumor) using an antibody and converts benign, therapeutically inactive prodrugs into active drug molecules directly at the site of action. This technique significantly reduces systemic drug distribution and allows creating higher local concentration of drugs, both phenomena contributing to an overall therapeutic benefit. Specificity of ADEPT greatly increases if the used enzyme is of non-mammalian origin (e.g. β-lactamase) or has a limited systemic distribution in a human body. In this initial investigation we chose to use β-glucuronidase (β-Glu) and glucuronide prodrugs as a system with adequate prior characterization. [37]–[40] Commercial availability of glucuronide prodrugs provides a further significant impetus for this choice. Finally, relatively large size of β-Glu enzyme (∼300 kDa) is also beneficial as it contributes to a higher retention of the enzyme within the hydrogel matrix.

To demonstrate implementation of SMEPT for drug delivery to cultured cells, we use µS PVA hydrogels as substrates for adhesion and proliferation of a model cell line, HepG2. Inasmuch as liver failure is among the leading causes of death worldwide, hepatic tissue engineering [30] attracts increased research attention and would significantly benefit from engineered substrate mediated controlled drug release. Furthermore, advanced efforts in engineering of liver tissue require co-culturing of mammalian cells (e.g. hepatocytes and Kupffer cells), [29], [31] a challenge which is successfully addressed using µS and micro-patterned substrates. [30], [31], [41] All of the above together justifies the use of a hepatic cell line and µS PVA hydrogels for the development of SMEPT.

In our recent work, [33] we developed enzyme equipped µS PVA hydrogels as surface adhered enzymatic micro-reactors. We focused on coagulation conditions as a tool to control materials properties of the matrix, namely polymer content and elasticity of the hydrogel, as well as retention of protein cargo and enzymatic activity of the latter. Developed method in localized conversion of the substrate within a hydrogel matrix was then adapted for conversion of benign prodrugs into therapeutically active product. Specifically, we presented initial demonstration of conversion of glucuronide derivative of SN-38, an anticancer drug, within β-Glu containing, cell adhesive PVA matrices, in the presence of adhered mammalian cells. This afforded an efficient cytotoxic effect and thus served as proof-of-concept illustration of SMEPT. In this work, we provide a detailed characterization of the system and reveal arms of control over this newly developed approach in substrate mediated drug delivery. In particular, we employ available tools of enzymatic catalysis to achieve control over the rate of substrate conversion over at least 3 orders of magnitude, illustrate performance of catalytically active matrices in cell media and in the presence of adhering cells, and analyze innate ability of model mammalian cells to perform conversion of glucuronide prodrugs into their respective products. With the use of glucuronide derivatives of SN-38, we demonstrate therapeutic effect and dose response achieved via SMEPT, i.e. external administration of prodrug at a desired concentration, its localized conversion, and internalization by adhered cells, which was comparable to that achieved via solution administration of pristine drug, SN-38. Taken together, these data contribute significantly to the development of hydrogels as intelligent biointerfaces equipped with innovative tools of drug delivery.

## Experimental Section

### Materials

Unless stated otherwise, all chemicals were purchased from Sigma-Aldrich and used without purification. Fluorescein di-glucuronide (FdG) and Presto Blue reagents were obtained from Invitrogen. SN-38 glucuronide was from Toronto Research Chemicals (Canada). DIC and fluorescence images were obtained using a Zeiss Axio Observer Z1 microscope; quantification of solution fluorescence was performed using an EnSpire Perkin Elmer multi-label plate reader; cells fluorescence was quantified using a BD-Accuri C6 flow cytometer.

### Methods

Assembly of surface adhered µS PVA hydrogels was performed as described previously. [33] In brief, 12 wt% PVA solution was heated to 50°C for 5 minutes to homogenize the solution and brought to 37°C for 5 minutes. An aliquot of PVA solution (typically 1,5 µL) was placed between a 9 mm glass cover slip and a poly(dimethylsiloxane) stamp with 2 µm cubic cavities and clamped at finger tight pressure for 24 h. Upon disassembly of the clamps, surface adhered µS thin films were stabilized for 1 h in a bath containing aqueous 0,5 M Na_2_SO_4_ and subsequently washed via immersion in PBS for 1 h.

For enzyme incorporation, PVA and β-Glu stock solutions were mixed via pipette assisted mixing at 37°C. Unless stated otherwise, final enzyme concentration in the polymer solution was 1 g/L. Unless stated otherwise, for evaluation of enzymatic activity, supernatants above µS hydrogels were exchanged to fresh PBS immediately before analysis, supplemented with FdG to 2,5 µg/mL concentration, allowed 30 minutes for enzymatic conversion with an end point evaluation of solution fluorescence using multi-label plate reader. All solution-based experiments were conducted at 37°C. Presented data are average of at least 3 independent experiments (3 replicates in each run) and presented as mean ± standard deviation.

For analysis of tools of control of drug release, µS PVA hydrogels were prepared following the above protocol and using mixtures of PVA with β-Glu with concentrations of enzyme 0,01, 0,1 and 1 g/L. For experiments performed at constant concentration of β-Glu, FdG stock solution was added to samples together with fresh PBS yielding prodrug concentrations of 2,5, 0,5, 0,05 and 0,025 µg/mL. In all cases, end-point evaluation of solution fluorescence was performed 30 minutes of incubation.

For analysis of protein loss from specimen, µS PVA hydrogels with incorporated enzyme were stabilized for 1 h with 0,5 M Na_2_SO_4_ and immersed into PBS or cell culture media for 1 h. Collected volumes of stabilization media, PBS and cell culture media were individually transferred to empty wells and tested for enzymatic activity by adding FdG stock solution to a final concentration of 0,25 µg/mL. µS samples were supplemented with PBS and then with FdG to a final concentration of 0,25 µg/mL. For all samples, enzymatic activity was evaluated via end-point evaluation of fluorescence after 30 minutes of incubation.

### Cell Culture

Hepatocellular carcinoma cell line HepG2 was purchased from Sigma-Aldrich. Cell culture was achieved according to the protocols provided by the manufacturer. µS PVA hydrogels were prepared on 9 mm round-shaped cover slips and placed into the wells of standard 48-well tissue culture plates. For cell culture experiments, µS PVA hydrogels were assembled using mixtures of PVA, β-Glu (1 g/L) and PLL (1 g/L) as described above. The cells were seeded at an initial density of 15,000 cells per well (analysis of SMEPT in the presence of adhering cells), 50,000 cells/well (fluorescence based experiments using FdG substrate) or 75,000 cells/well (cell viability experiments) and allowed 24 h for attachment with exchange of media prior any further evaluation or treatment. For comparison of solution based enzyme prodrug therapy and SMEPT, solution conversion was achieved using 1,5 µg of β-Glu added to PBS (experiments with FdG) and cell culture media (SN-38 glucuronide). For analyses in flow cytometry, at specified time points the cells were harvested using trypsin (0,05% trypsin, 3–5 minutes) and stored in PBS on ice. For cell viability assays, µS PVA hydrogels were fabricated following protocols described above. Unless stated otherwise, SN-38 or SN-38 glucuronide were added to yield a concentration of 1 µM and incubated with cultured cells for 48 h. Cell viability was quantified using Presto Blue reagent following manufacturer’s protocols and a 30 minutes of incubation time.

## Results and Discussion

**Figure 2:**
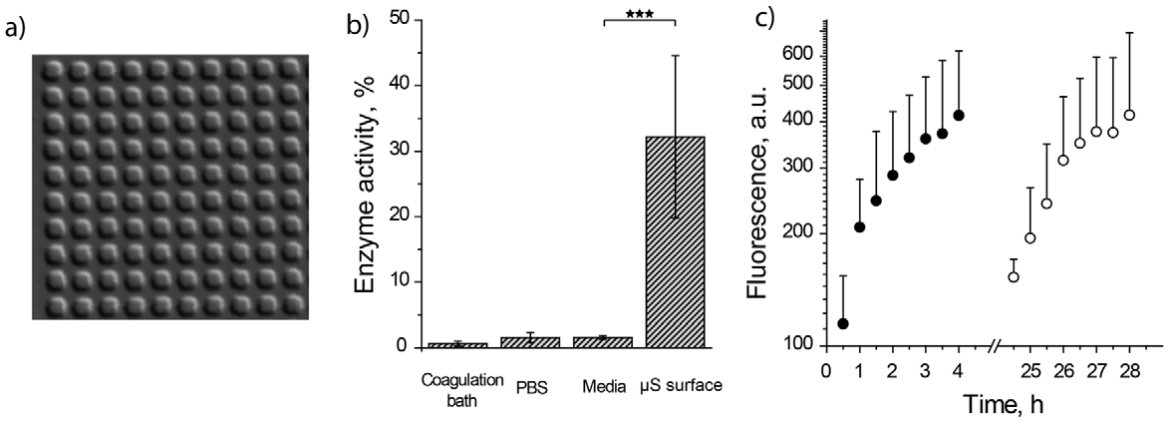
Visualization and initial characterization of µS PVA hydrogels as matrices for SMEPT. (a) Differential interference contrast microscopy image of the surface adhered µS PVA hydrogels (2 µm cubic structures). (b) Levels of β-Glu enzymatic activity revealed by polymer coagulation bath, PBS washing solution, cell culture media washing solution, and final surface adhered µS PVA hydrogels. Enzyme quantification was performed using FdG, 30 min reaction time and a standard curve obtained for the activity of β-Glu in PBS. (C) Experimental data for conversion of FdG into fluorescent product using µS PVA hydrogels and initiating conversion via administration of FdG at time points t = 0 (solid circles) and t = 24 h (open circles). Experimental conditions: [FdG]: 2,5 µg/mL; 1 g/L β-Glu in the polymer solution used for the production of hydrogels.

µS PVA hydrogels were assembled via micro-transfer molding (µTM) using solutions of PVA mixed with β-Glu, PDMS stamps with 2 µm-side cubic cavities, and sodium sulfate as a polymer coagulating salt, [23], [24] Figure 2,a. In all experiments, to ensure identical surface area of the hydrogels and minimize sample-to-sample variation, µTM was performed using 9 mm cover slips and PDMS stamps with exceeding dimensions. Solutions of mixtures of PVA with the enzyme were prepared via a simple pipette assisted mixing of polymer and enzyme stock solutions, which ensures a facile, convenient way to adjust the concentration of either component through the choice of mixed volumes or concentrations of the stocks. Fig. 1,a demonstrates that the presence of β-Glu at a 1 g/L final concentration does not interfere with polymer gelation and resulting samples are robust and stable in a hydrated state in PBS.

For sensitive and quantitative analysis of enzymatic activity, we employed a di-glucuronide derivative of fluorescein (FdG), a non-fluorescent substrate, enzymatic conversion of which yields a highly fluorescent product. To verify incorporation and retention of the enzyme in the hydrogels, enzymatic activity was quantified in collected volumes of polymer coagulation bath, subsequent PBS washing solution, as well as in hydrogel preparations immersed in PBS. To achieve this, samples were charged with FdG to a final concentration of 0,25 µg/mL and allowed 30 min for enzymatic conversion of FdG into fluorescein after which solution fluorescence was quantified using a multi-label plate reader. Using calibration curve, values of fluorescence intensity were converted into levels of enzymatic activity relative to β-Glu in an amount equivalent to that used for µTM and analyzed in PBS. Coagulation bath and PBS washing solution revealed minimal levels of enzymatic activity, Figure 2,b. In contrast, µS hydrogels afforded a pronounced level of conversion of FdG into a fluorescent product with at least a 30% level of β-Glu activity. With cell culture application as a final goal, we also quantified enzymatic activity in cell culture media wash aspirated from µS hydrogels, and this too revealed only a minor level of fluorescence, i.e. minimal enzymatic activity. Presented data indicate that µS hydrogels prepared via µTM and using non-cryogenic gelation technique retain a significant amount of the enzyme in their structure, a notion which comes in contrast with conventional, cryogenic PVA hydrogels. For the latter, protein conjugation to PVA or anchoring macromolecules is typically required to ensure immobilization and retention of the enzymatic cargo. [42], [43] Large size of β-Glu enzyme is thought to contribute significantly to the observed level of cargo retention. We also note that the data in Figure 2,b do not rule out loss of the enzyme from the hydrogel phase and it is highly probable that a fraction of incorporated protein does escape into solution bulk. For interpretation of the data presented below, it is imperative that while collected supernatant solutions exhibited non-negligible levels of enzymatic activity, these are at least 10-fold lower than enzymatic activity mediated by the hydrogels, a notion which implies that an overall majority of enzymatic conversion occurs within the hydrogel phase.

To further verify retention of the enzyme within the hydrogels, we used two identical samples of enzyme-loaded µS PVA thin films and recorded kinetics of conversion of FdG into the fluorescent product having administered FdG in two samples 24 h apart, Figure 2,C. The revealed rates of presentation are near identical which implies a similar content of the enzymatic cargo within the hydrogel phase, i.e. a minimal loss of enzymatic cargo from µS PVA hydrogels during a 24 h incubation in PBS or inactivation of the immobilized enzyme. This data allows making a further important conclusion, namely that SMEPT is devoid of “burst release” phenomenon, the latter being a persistent shortcoming of drug eluting matrices and hydrogels in particular. [7] Indeed, in contrast to existing tools in controlled drug release, for SMEPT, immersion into a test milieu produces no active product and drug release is initiated at the moment of choice by administration of the pro-drug. Further to this, drug release via SMEPT is subject to external control and not engineered into the matrix, as is further detailed by the following experiments.

**Figure 3:**
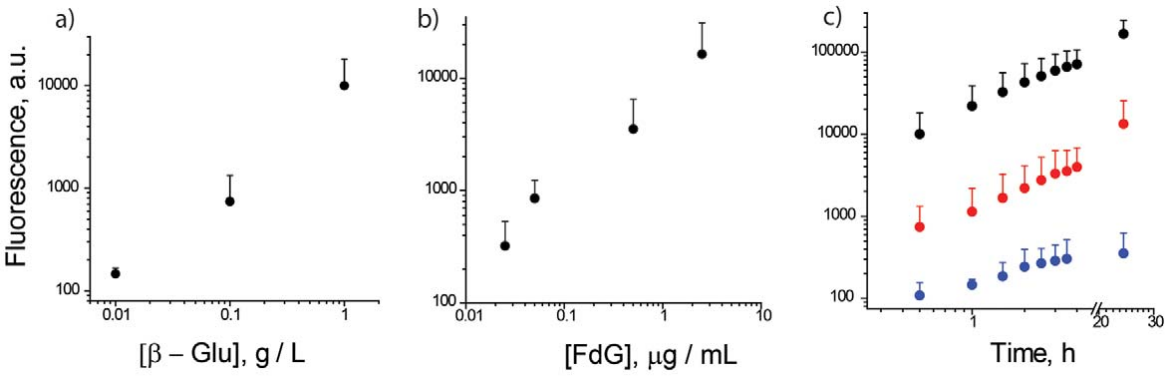
SMEPT offers several arms of control over the rate of generation and the overall amount of the product generated by the enzyme containing substrates: concentration of the enzyme in the gel (a), concentration of the added substrate (b) and time of enzymatic conversion (c). Experimental conditions: (a) FdG, 2,5 µg/mL, 30 min reaction time; (b): 1 g/L β-Glu in polymer solution, 30 min reaction time; c) FdG: 2,5 µg/mL; β-Glu: 1 (top), 0.1 (middle) and 0.01 (bottom) g/L, respectively. Presented results (mean ± st.dev.) are average over at least 3 independent experiments, 3 replicates each.

By design, flexible and adaptable nature of SMEPT allows controlling the rate of drug presentation and the overall quantity of the generated product by several independent methods, as is well documented in the field of enzymatic catalysis. At a constant concentration of a prodrug, generation of the product can be rationally programmed by the concentration of the protein within the µS PVA thin film. To demonstrate this, µS hydrogels were prepared as described above using mixtures of PVA with β-Glu with concentration of the latter varied over 2 orders of magnitude. Resulting hydrogels were immersed in a volume of PBS which was then supplemented with FdG to a 0,25 µg/mL concentration and the system was allowed 30 minutes to perform enzymatic conversion. After this, fluorescence of aspirated supernatants was quantified using multi-label plate reader, Figure 3,a. Higher enzyme content achieves a faster conversion of the substrate, and the end-point analyses reveal progressively higher levels of solution fluorescence attained with increased content of the enzyme in the gel. A single administered dose of a prodrug can therefore yield a judiciously chosen concentration of the product at the site of action varied over at least 2 orders of magnitude. Importantly, a 100-fold increase in the enzyme content within a mixture with PVA affords µS hydrogels with ∼100-fold change in the level of experimentally determined enzymatic activity which reveals a superior control over the rate of product synthesis and release achieved through a variation of protein content in a SMEPT matrix.

At a given enzyme loading, the rate of product presentation is conveniently determined by the concentration of administered prodrug (Figure 3,b). To illustrate this opportunity, µS PVA hydrogels were prepared with a constant concentration of β-Glu and allowed 30 min for enzymatic conversion in PBS supplemented with FdG to a varied concentration, from 0.025 to 2,5 µg/mL (∼0.035÷3.5 µM). This range of concentrations is well below typical Km values for β-Glu (18 µM–1.1 mM), [44]–[46] and the end point analyses of substrate mediated enzymatic reactions reveal a linear increase in solution fluorescence with increased concentration of FdG. As with variation of enzymatic conversion through the choice of protein concentration, this result as such is rather expected. However, to the best of our knowledge, these arms of control have not been previously adopted for surface mediated controlled drug release.

While results presented above reveal arms of control over the rate of product synthesis, total deliverable payload achieved by an individual µS PVA hydrogel sample is further controlled by the time of enzymatic reaction, Figure 3,c. At each concentration of the enzyme, increased conversion time allows a greater fraction of FdG be converted into the final product, as evidenced by increased intensity of solution fluorescence. We emphasize that in situ generation of the product by a catalytic enzyme affords a dramatic amplification of a deliverable payload and 10^−14^ mole enzyme produces at least ∼µM concentration of the product (Figure 3,c). Furthermore, the total deliverable payload is not a set value engineered into the matrix, as is typically the case for drug eluting implants, but is a subject to continuous interactive modulation. Taken together, we believe that the above data reveal significant promise of SMEPT for surface mediated delivery.

To verify utility of SMEPT for drug delivery to adhering cells, we employed a model hepatic cell line, HepG2, and used µS PVA hydrogels as substrates for cell adhesion and proliferation. While pristine PVA is a low fouling polymer and affords non cell adhesive hydrogels, [27] we have previously shown that supplementing PVA solution with poly-L-lysine and co-gelation of the two polymers results in matrices which are well suited for cell culture, [23], i.e. support adhesion and proliferation of mammalian cells. In separate experiments we confirmed that the presence of PLL does not result in drastic changes in the levels of conversion of FdG into fluorescein mediated by β-Glu.

**Figure 4:**
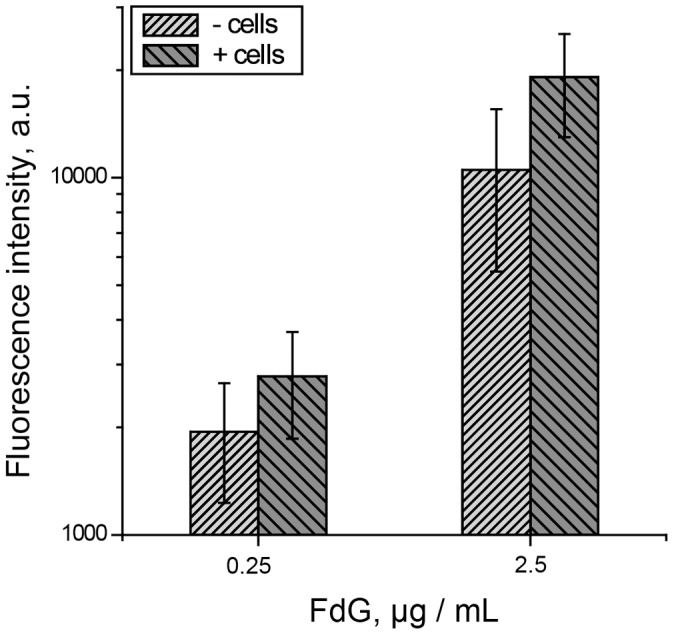
Conversion of the prodrug by the enzyme within a PVA hydrogel matrix is not impaired by the presence of serum, i.e. possible absorption of proteins, and adhesion of mammalian cells. Experimental conditions: 1 g/L β-Glu in polymer solution, 30 min reaction time. Presented results (mean ± st.dev.) are average over at least 3 independent experiments, 3 replicates each.

A plausible limitation to the performance of SMEPT lies in that adhering cells, as well as adsorbed serum proteins, may hinder diffusion of solutes through the hydrogel interface and in doing so arrest exchange of the (pro)drugs between the hydrogel and solution bulk. To probe this, HepG2 cells were seeded on µS PVA surfaces and allowed 24 h for initial adhesion and proliferation. Following exchange of media, a step which was taken to ensure removal of the protein plausibly released from the hydrogels, fluorogenic substrate was administered onto the cultured cells and fluorescence of solutions was quantified upon a 30 minute conversion time. For the two tested concentrations of FdG, production of fluorescent cargo was not impaired by adhering cells, as evidenced by similar levels of solution fluorescence, Figure 4. In fact, registered fluorescence was slightly higher for the supernatants above µS PVA substrates with adhered cells as compared to the cell-free matrices. While no attempt was made in this work to culture cells to their confluence and attempt to deliberately block solute access to the hydrogels, presented results demonstrate that performance of SMEPT in routine cell culture is not impaired by the presence of serum or cell adhesion.

**Figure 5:**
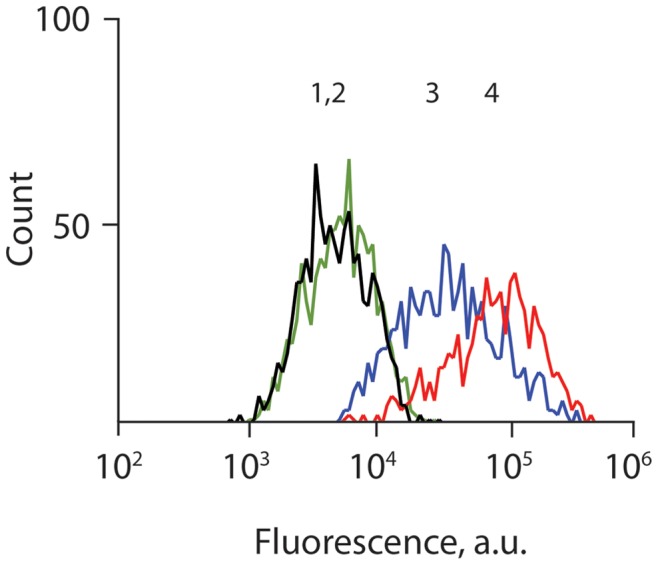
Utility of SMEPT in conversion of fluorogenic prodrug and cargo uptake is verified through quantification of fluorescence of hepatic cells cultured on the µS PVA hydrogels. Administration of FdG in the absence of enzyme led to a negligible change in the fluorescence of cultured cells (traces 1 : cells only, trace 2 : +FdG). SMEPT conditions (trace 3 : β-Glu immobilized within µS hydrogels, +FdG) afforded comparable level of fluorescence of the cultured cells as solution based administration (trace 4 : β-Glu and FdG are added to media above cultured cells), as quantified by flow cytometry analysis of harvested cells. In all cases, [FdG] = 2,5 µg/mL.

In the next experiment, we compared generation and internalization of a fluorescent reporter molecule produced via SMEPT and via solution-based conversion, Figure 5. Towards this end, β-Glu containing matrices were prepared as described above and used as substrates for adhesion of HepG2 cells and matrices for conversion of FdG into fluorescein. In a separate experiment, HepG2 cells were cultured on enzyme-free µS PVA hydrogels in media supplemented with β-Glu and FdG, i.e. solution based enzyme prodrug therapy (EPT). Further control included HepG2 cells cultured in media in the presence of FdG and in absence of β-Glu in solution or within cell adhesion matrix. The latter conditions were employed to reveal possible levels of inherent glucuronidase activity within these cells. In all cases, fluorescence of the harvested cells was analyzed following 1 h incubation in the presence of FdG using flow cytometry, Figure 5. Administration of the prodrug in the absence of enzyme in solution or within the gel phase afforded no fluorescent product and the cells exhibited fluorescence identical to pristine, non-treated cells (Figure 5, traces 1,2). This notion demonstrates a low level of non-specific, inherent conversion of glucuronide prodrugs by hepatic cells. In contrast, SMEPT conditions afforded levels of cellular fluorescence comparable to that attained when using the enzyme and the prodrug in solution based administration, in both cases significantly higher than fluorescence of pristine cells. We note that solution based prodrug conversion was used herein only to verify protein activity and utility of SMEPT. In humans, β-Glu has a limited spread and activity markedly lower as compared to bacterial copy used in this study. [34], [35] The latter notion provides for a low level of “background” prodrug conversion and contributes to specificity of drug delivery achieved with the use of β-Glu and glucuronide prodrugs, as documented for ADEPT [34], [35] and inherited by SMEPT.

**Figure 6:**
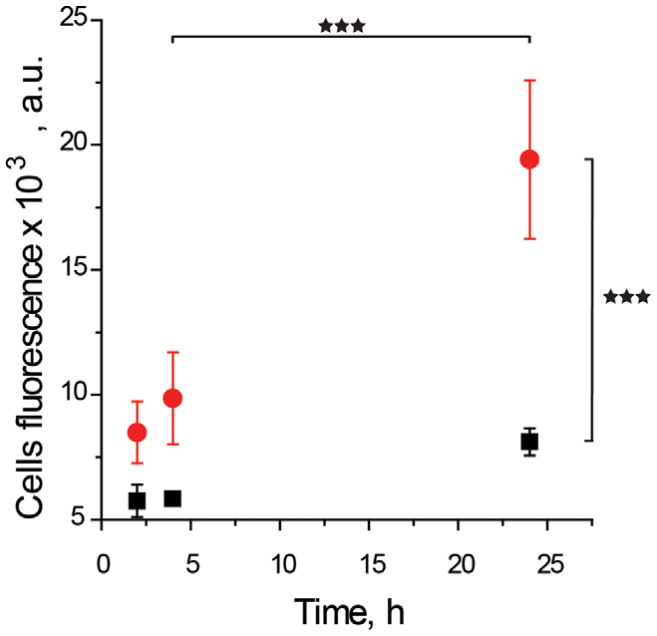
Time- and dose- dependent cellular internalization of the model fluorescent product, fluorescein, generated via SMEPT from its prodrug, FdG. Experimental conditions: 1 g/L enzyme in the polymer solution; initial concentration of FdG: 0.25 (red circles) and 0.025 (black squares) µg/mL. Presented results (mean ± st.dev.) are average over at least 3 independent experiments, 3 replicates each.

For further characterization of SMEPT, we followed the time course of cellular internalization of in situ generated fluorescent product. For these experiments, µS PVA hydrogels were prepared using mixtures of PVA with β-Glu at a final protein concentration of 1 g/L. Following initial cell adhesion (24 h) and exchange of media, samples were supplemented with FdG to concentrations of 0.025 and 0.25 µg/mL (36,5 and 365 nM, respectively), and fluorescence of harvested cells was quantified at specified time points using flow cytometry, Figure 6. A pronounced increase in the fluorescence of cells was registered already at the earliest time-point, 2 h. With increased time, the cells exhibited progressively higher levels of fluorescence and this provides evidence of continuous enzymatic conversion of the prodrug and internalization of the reporter cargo. Further to this, at each time point, higher prodrug concentration affords a higher level of fluorescence registered in the cultured cells, i.e. a greater amount of internalized cargo. These data demonstrate that tools of enzymatic catalysis used in the context of SMEPT (Figure 3) are also successfully implemented for controlled delivery of model fluorescent cargo to the cultured cells.

**Figure 7:**
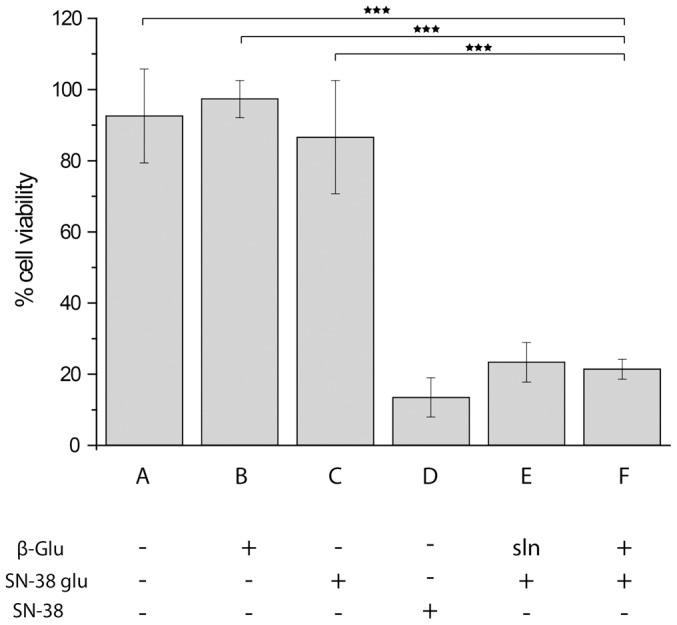
Viability of HepG2 cells cultured on µS PVA thin films as quantified using Presto Blue viability assay and expressed relative to viability of these cells on tissue culture polystyrene at a matched initial cell seeding density. The cells were cultured on (A) pristine µS PVA hydrogels; (B) µS PVA thin films equipped with β-Glu; (C) enzyme-free µS PVA hydrogels in the presence of 1 µM SN-38 glucuronide; (D) enzyme-free µS PVA hydrogels, 1 µM SN-38; (E) µS PVA hydrogels in the presence of 1 µM SN-38 glucuronide and β-Glu added to the cell media (solution based enzyme prodrug therapy); (F) SMEPT conditions, i.e. β-Glu equipped µS PVA thin films in the presence of 1 µM SN-38 glucuronide. Presented results (mean ± st.dev.) are average over at least 3 independent experiments, 3 replicates each.

To quantify therapeutic effect achieved via SMEPT, we employed a glucuronide prodrug of a potent anticancer agent, SN-38 (SN-38-glu). In preliminary experiments we verified that this metabolite of irinothecan is characterized with sub-micromolar IC_50_ and requires 48 h incubation with HepG2 to manifest optimal activity. µS PVA hydrogels were prepared as described above and used as substrates for adhesion of HepG2 cells with 24 h allowed for an initial adhesion of cells following an exchange of media and 48 h incubation with the (pro)drug at a 1 µM concentration. Resulting metabolic activity of the cells was quantified using Presto Blue cell viability reagent and expressed in Figure 7 relative to the viability of cells cultured on tissue culture polystyrene multi-well plates at identical cell seeding density. In the absence of administered (pro)drug, viability of the cells cultured on µS PVA remained un-altered (sample A), an observation which verifies biocompatibility of PVA hydrogels for cell adhesion. For SMEPT, the matrices were equipped with β-Glu (1 g/L protein concentration in PVA solution employed in µTM). Resulting µS surfaces sustained a near 100% metabolic activity of the adhering cells (sample B), a notion which substantiates utility of the developed matrices for SMEPT. As expected, addition of SN-38-glu in the absence of β-Glu led to an insignificant decrease in cell viability (sample C). In turn, addition of the parent drug, SN-38, resulted in a decrease in cell viability to 20% (sample D), which is similar to that observed at this concentration of the drug on the cells cultured on tissue culture polystyrene (data not shown). Solution-based enzyme prodrug therapy, i.e. administration of SN-38-glu together with the enzyme to the supernatant above the cultured cells, elicited a similar therapeutic response, i.e. a decrease in cell viability to 20% (sample E). Finally, administration of the prodrug to the cells cultured on the enzyme-equipped PVA matrices, i.e. SMEPT conditions, afforded a near identical therapeutic response and a decrease in cell viability to 25% (sample F) thus demonstrating therapeutic effect achieved via SMEPT. We note that this data set also demonstrates that SMEPT is not unique to a single prodrug (e.g. FdG) but can be applied to glucuronide derivatives of diverse cargo, therapeutic or fluorescent, to achieve therapeutic response or for visualization purposes, speaking towards adaptability and general utility of this concept.

**Figure 8:**
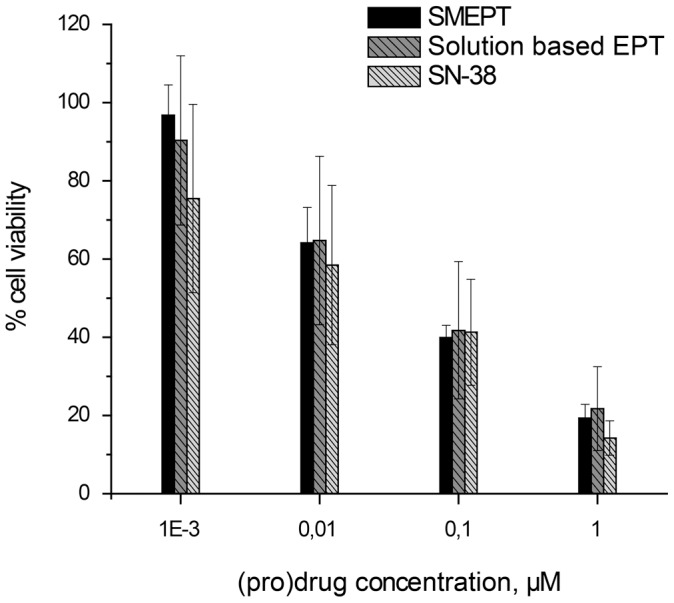
Dose response for HepG2 cells to the administered SN-38 glucuronide (for SMEPT and solution based EPT) or SN-38. Presented results (mean ± st.dev.) are average over at least 3 independent experiments, 3 replicates each.

To further demonstrate arms of control associated with drug delivery via SMEPT, we performed a dose response experiment and compared cell viability as attained using pristine SN-38, solution based enzyme prodrug therapy, and SMEPT, Figure 8. At each concentration within the tested range, from 1 nM to 1 µM, the three drug administration approaches afforded similar therapeutic effects. In other words, enzymatic conversion of the prodrug was successfully achieved by the enzyme added to the media (solution based EPT) as well as by the enzyme within the hydrogel structure, i.e. SMEPT. Matched cell viabilities imply that the drug was generated at concentrations similar to administered SN-38 and therefore suggest a near quantitative conversion of the prodrug into an active therapeutic. We emphasize that for each experiment presented in Figure 8, SMEPT methodology uses the same amount of immobilized enzyme, and controlled drug dosage was achieved via a judicious choice of administered pro-drug. In contrast to typical drug eluting matrices, drug dosage was controlled externally, not engineered into the matrix, revealing that SMEPT combines benefits of “conventional” drug administration, facile fine-tuning of drug regimen, and surface mediated drug release, i.e. localized presentation of therapeutic cargo.

### Conclusions

Taken together, experiments presented above present substrate mediated enzyme prodrug therapy as a novel tool of drug delivery. We envision that SMEPT is highly adaptable and is not limited to particular enzyme, prodrugs, and methods of immobilization used in this study. We anticipate that SMEPT will find use in diverse biotechnological and biomedical applications, specifically surface mediated drug delivery [32] and tissue engineering. [15], [47], [48]. We are now investigating long-term performance of SMEPT and its utility in site-specific delivery of anti-inflammatory and anti-viral therapeutics.
